# Absence of methicillin-resistant *Staphylococcus aureus* colonization among immunocompetent healthy adults: Insights from a longitudinal study

**DOI:** 10.1371/journal.pone.0253739

**Published:** 2021-06-30

**Authors:** Sónia T. Almeida, A. Cristina Paulo, João Babo, João Borralho, Catarina Figueiredo, Bruno Gonçalves, João Lança, Mónica Louro, Hermes Morais, Joana Queiroz, Hermínia de Lencastre, Raquel Sá-Leão

**Affiliations:** 1 Laboratory of Molecular Microbiology of Human Pathogens, Instituto de Tecnologia Química e Biológica António Xavier, Universidade Nova de Lisboa, Oeiras, Portugal; 2 Laboratory of Molecular Genetics, Instituto de Tecnologia Química e Biológica António Xavier, Universidade Nova de Lisboa, Oeiras, Portugal; 3 Laboratory of Microbiology and Infectious Diseases, The Rockefeller University, New York, NY, United States of America; Universidade de Lisboa Faculdade de Medicina, PORTUGAL

## Abstract

Methicillin-resistant *Staphylococcus aureus* (MRSA) has long been known as a major cause of hospital-acquired (HA-MRSA) infections worldwide. For the past twenty years, an increasing number of studies have described its emergence in the community as well. In Portugal, a country with a high-prevalence of HA-MRSA, there are only limited data available on the epidemiology of MRSA in the community. We studied the prevalence of *S*. *aureus* and MRSA colonization among healthy adults in Portugal. Between February 2015 and December 2016, a longitudinal study was conducted in which 87 adults aged 25–50 years old were followed for six months. For each participant nasopharyngeal, oropharyngeal and saliva samples were obtained monthly and, in some cases, weekly. A total of 1,578 samples (n = 526 for each sampling site) were examined for the presence of *S*. *aureus* and MRSA by classical culture-based methods. Fifty-seven adults (65.5%) carried *S*. *aureus* at least once during the six months period of the study: 19.5% were persistent *S*. *aureus* carriers and 46.0% were intermittent carriers. Carriage rates per sampling site were 20.5% in nasopharynx, 18.3% in oropharynx, and 13.5% in saliva. Simultaneous screening of the three sampling sites increased detection of *S*. *aureus*, which overall occurred in 34.4% of the 526 sampling time-points. No MRSA were isolated. In conclusion, this study adds novel information about the MRSA scenario in the Portuguese community. Our results indicate that, in Portugal, MRSA does not seem to circulate among healthy adults without risk factors and therefore this age group does not constitute, at the current time, a reservoir of MRSA in the community.

## Introduction

*Staphylococcus aureus* is a common colonizer of the human anterior nares. About 20%-40% of the general population is colonized with this bacterium. It is also an important pathogen that is responsible for both health-care and community infections, such as skin and soft tissue infections, pneumonia, endocarditis and bacteremia [1, 2].

Methicillin-resistant *S*. *aureus* (MRSA), in particular, is responsible for high rates of nosocomial infections worldwide and in the last two decades, has also emerged and spread in the community (community-associated MRSA, CA-MRSA) worldwide [3–7].

In recent years, although the rates of hospital-associated MRSA (HA-MRSA) have decreased in most European countries, including Portugal, this pathogen continues to be a serious cause of bacterial infections. In Portugal, the prevalence of HA-MRSA, among all *S*. *aureus* obtained from blood and cerebrospinal fluid, is the third highest in Europe having been estimated as 38.1% in 2018 [6].

In Portugal, national surveillance studies have been conducted for almost 30 years in order to follow the prevalence of HA-MRSA over time. In the early 1990s the dominant clone in the Portuguese hospitals was the Iberian clone (ST247-IA) that was replaced by the Brazilian clone (ST239-IIIA) in 1995. In 2001, the EMRSA-15 clone emerged in the country and soon became the dominant clone in most hospitals. Today, it still remains one of the most prevalent clones [8, 9].

Despite the emergence of CA-MRSA infections worldwide, studies among healthy populations suggest that carriage rates remain low in most parts of the world. Cross-sectional analysis among USA adults aged between 20–49 years old and Queensland adults aged between 18- >59 showed a prevalence of CA-MRSA nasal colonization of 0.8% and 0.7%, respectively [10, 11]. In Europe, studies from Ireland, Malta, and Greece estimated that the prevalence of CA-MRSA ranged between 0.7%-5.2% among adults aged between 16–60 years old [12–14]. In addition, a longitudinal study conducted among the German general population (aged between 7–97 years old) also showed very low (0.7%) rates of MRSA [15]. Furthermore, colonization rates among senior adults aged ≥65 years old living in Germany and Brazil were similar to the ones described for younger adults, 0.7% and 3.7%, respectively [16, 17].

In Portugal, although several studies have been conducted in the nosocomial setting, less is known about the epidemiology of MRSA in the community. Previous screenings of MRSA among young adults, such as draftees (aged 17–22 years old), non-medical university students (aged 21–24 years old) and high-school students (aged 13–16 years old), and among the elderly reported a very low prevalence of MRSA carriage, 0.7% and 1.8%, respectively [18, 19]. In addition, a carriage study conducted in children up to 6 years old attending day-care centers, also showed a very low (0.2%) prevalence of MRSA in the nasopharynx [20].

Regular surveillance studies are needed to monitor and prevent dissemination of potential pathogens–such as MRSA—and adapt strategies to prevent infections. To our best knowledge, in Portugal, MRSA colonization studies among immunocompetent healthy adults have not been performed before, and it is unknown whether this age group may constitute a reservoir of MRSA in the community.

The aim of this study was to evaluate the prevalence of asymptomatic colonization of *S*. *aureus* and MRSA in the community among immunocompetent healthy adults aged between 25–50 years old, living in Portugal.

## Materials and methods

The study was approved by the ethical committee of Instituto de Higiene e Medicina Tropical, Universidade Nova de Lisboa and was registered at National Commission of Data Protection (ref. 3803/2014). Signed informed consent was obtained from all participants; samples and questionnaires were processed anonymously.

In order to conduct this work, we took advantage of a longitudinal study that our group conducted previously, among adults aged between 25–50 years old. The details of the study design have been described previously [21]. Briefly, between February 2015 and December 2016, 87 adults aged 25–50 years old, living in the Lisbon area, were followed for 6 months. For each participant nasopharyngeal, oropharyngeal and saliva samples were obtained monthly. In some cases, individuals were sampled weekly. For the purpose of this study, we focused on sampling time-points in which the three types of samples were obtained. Overall, 1578 samples (526 nasopharyngeal samples, 526 oropharyngeal samples and 526 saliva samples) were analyzed.

Nasopharyngeal samples were collected using a flexible swab with a flocked nylon fiber tip (reference 482CE from Copan) and oropharyngeal samples were collected with a rigid swab with a flocked nylon fiber tip (reference 480CE from Copan) as described previously [21]. Saliva was collected by spitting into a tube. 50μl of each sample were plated onto mannitol salt agar (Difco, Detroit, MI) and incubated in aerobic conditions, overnight at 37°C. On the following day, one mannitol-positive colony was streaked onto tryptic soy agar (Difco) and incubated overnight at 37°C. All presumptive *S*. *aureus* cultures were tested for coagulase production using the latex agglutination test Staphaurex (Remel, Lenexa, KS).

Samples considered to be *S*. *aureus* positive were tested for cefoxitin susceptibility using agar disk diffusion, according to the Clinical and Laboratory Standards Institute (CLSI) guidelines [22]. *S*. *aureus* isolates displaying an inhibition zone against cefoxitin ≤21 mm were considered to be putative MRSA.

Persistent *S*. *aureus* carriers were defined as individuals with at least three consecutive positive monthly samples. Intermittent carriers were defined as individuals carrying *S*. *aureus* with less than three consecutive monthly positive samples. Non-carriers were defined as individuals from which *S*. *aureus* was never recovered.

To compare the prevalence of *S*. *aureus* between nasopharyngeal, oropharyngeal and saliva samples the McNemar’s test was used. A p-value of <0.05 was considered statistically significant. All statistical analysis was performed using R version 3.6.2 [23].

## Results

A total of 87 adults between the ages of 25–50 years old, living in the Lisbon region, participated in this study. The characteristics of the population are described in Table 1. Briefly, the mean age of the participants was 37.1 ± 6.4 years and 49.4% were female. More than half of the participants (57.5%) lived with children under 18 years old, while few participants (6.9%) lived with adults aged ≥65 years old. A total of 43.7% of the participants were smokers and 51.7% were exposed to smoke. Hospitalization within the six months preceding enrollment was low with 2.3% of the participants reporting previous hospitalization (Table 1).

**Table 1:** Socio-demographic characteristics of the participants.

Variable	Participants (total = 87)
Mean age (years)	37.1 ± 6.4
<40 years old	40.2% (52)
≥40 years old	59.8% (35)
Gender	
female	49.4% (43)
male	50.6% (44)
Body mass index (kg/m^2^)^a^	
normal weight	60.9% (53)
underweight	2.3% (2)
overweight	36.8% (32)
Household size	
≤2	58.6% (51)
>2	41.4% (36)
Living with adults ≥65 years	6.9% (6)
Living with children (≤ 18 years)	57.5% (50)
Smoker	43.7% (38)
No. of years as smoker	
≤15	19.5% (17)
>15	24.1% (21)
No. of cigarettes per day	
≤15	25.3% (22)
>15	29.9% (26)
Smoke exposure^b^	51.7% (45)
Chronic diseases^c^	26.4% (23)
Long term medication^d^	23.0% (20)
Seasonal flu vaccination	6.9% (6)
Pneumococcal vaccination	8.0% (7)
Pneumococcal vaccination with PCV13	6.9% (6)
Pneumococcal vaccination with PPV23	1.1% (1)
At enrollment	
Antibiotic consumption within the 6 months preceding enrollment	19.5% (17)
Hospitalization within the 6 months preceding enrollment	2.3% (2)
Disease within the 6 months preceding enrollment^e^	9.2% (8)
Antibiotic consumption at least once during the 6-month follow-up	24.1% (21)

^a^Body mass index calculated as weight/height^2^ and classified according to WHO as underweight if BMI<18.5, normal weight if 18.5≤BMI≤24.9, and overweight if BMI≥25

^b^at home (n = 8), at the working place (n = 20), by a partner who smoke (n = 23), independently of being a smoker

^c^sinusitis (n = 10), asthma (n = 3), allergic rhinitis (n = 2), heart diseases (n = 2), bronchiectasis, hypertension, hypothyroidism, obesity, neurological diseases and psoriasis (n = 1 each)

^d^oral contraceptives (n = 12), α-blockers for hypertension (n = 4), antihistamines (n = 1) and medication for hypothyroidism (n = 1), venous insufficiency (n = 1), asthma (n = 1) and psychiatric disorders (n = 1)

^e^respiratory infections (n = 4), gynecologic disorders, cutaneous infection, urinary tract infection and blunt trauma (n = 1 each).

During the six months of the study, from the estimated 522 (87x6) monthly sample time-points, 455 (87.2%) were obtained. In addition, there were 145 weekly sampling time-points that occurred given the original study design, aiming to closely monitor the dynamics of carriage of *Streptococcus pneumoniae*. Overall, there were a total of 600 sampling time-points. Of these, in 526 the three types of samples (nasopharynx, oropharynx and saliva) were obtained, yielding 1,578 samples (526 each), all of which were screened for the presence of *S*. *aureus* and MRSA. In the remaining 74 sampling time-points, the three types of samples were not obtained and, therefore, were not analyzed in this study.

Prevalence of *S*. *aureus* by sampling site is summarized in Table 2. Among the 1578 samples screened, 275 samples (17.4%) were positive for *S*. *aureus*. There were no significant differences in the detection of *S*. *aureus* when comparing the nasopharynx with the oropharynx: 20.5% vs. 18.3% (p = 0.306), respectively. In contrast, *S*. *aureus* was more frequently detected in nasopharyngeal samples or oropharyngeal samples than in saliva samples: 20.5% vs. 13.5% (p<0.001) and 18.3% vs. 13.5% (p = 0.01), respectively. Of note, 17.2% (n = 15) of the participants carried *S*. *aureus* in the three sampling sites simultaneously, at least once.

**Table 2:** Detection of *S*. *aureus* according to the sampling site.

Sampling site	No. of isolates (%)
Nasopharynx (n = 526)	108 (20.5%)
Oropharynx (n = 526)	96 (18.3%)
Saliva (n = 526)	71 (13.5%)

**Fig 1:**
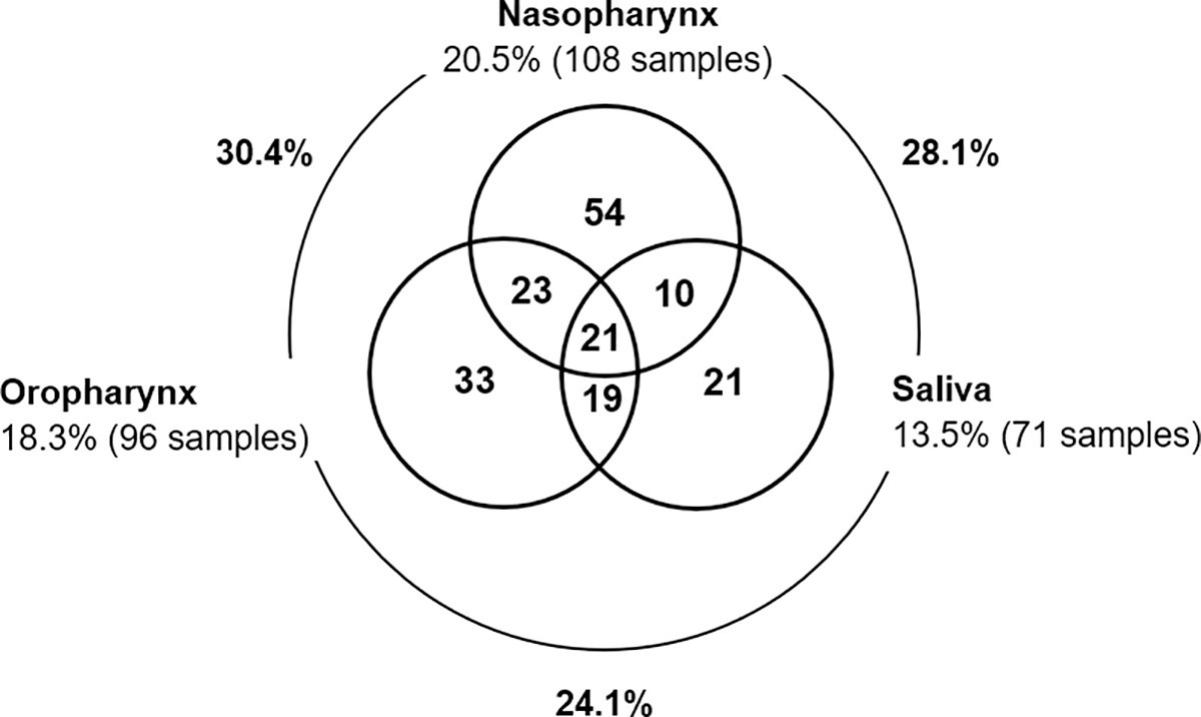
Positive sites for *S*. *aureus* among the 526 sampling time-points in which three sampling sites (nasopharynx, oropharynx, and saliva) were screened. There were 345 sampling time-points in which the three sampling sites were negative for *S*. *aureus*. Each circle represents the indicated sampling site. The numbers inside circles indicate the number of positive samples for *S*. *aureus*. Overlapping areas indicate the number of positive samples in which simultaneously detection of *S*. *aureus* in more than one sampling site occurred. Percentages of positive samples are indicated, as well as concordance between sampling sites.

Simultaneous sampling of the three sites led to detection of *S*. *aureus* carriage events in 34.4% of the 526 sampling time-points. Sampling both nasopharynx and oropharynx allowed detection of virtually all positive samples (30.4%) (Fig 1).

**Fig 2:**
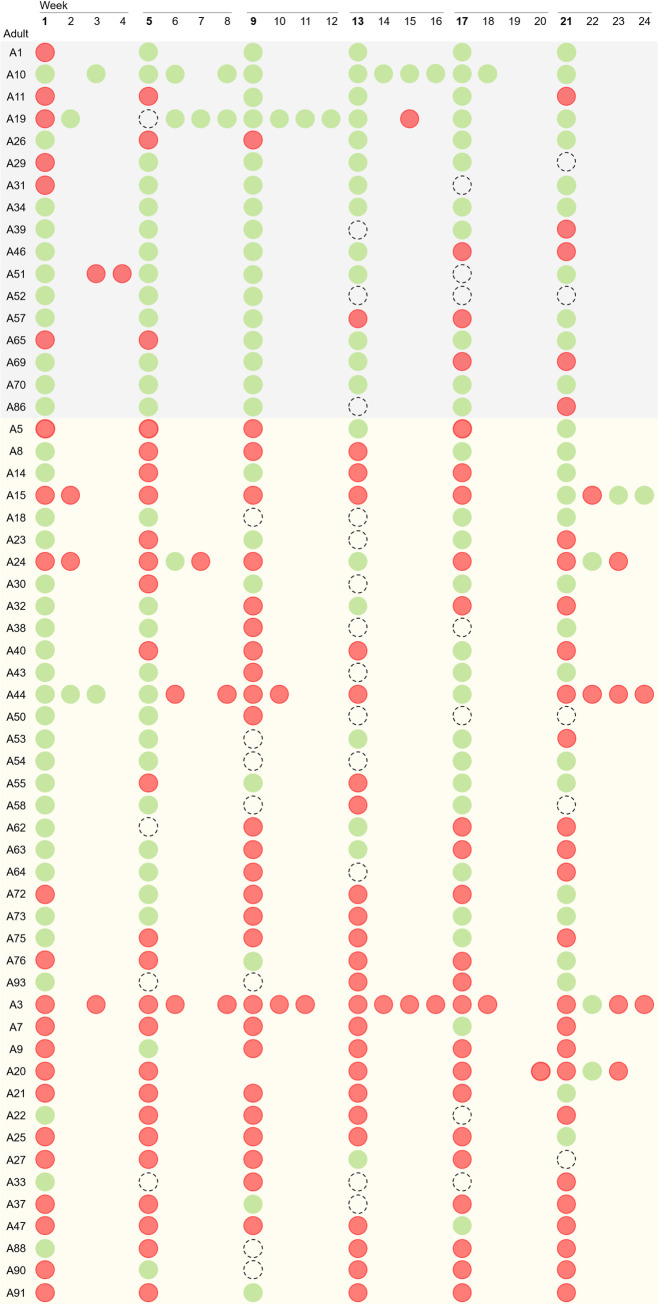
*S*. *aureus* carriage dynamics of the 57 participants that were colonized at least once during the six months of the study. Red circles represent negative samples; green circles represent positive samples for *S*. *aureus*; dotted circles represent expected samples (as per protocol) that were not obtained. The grey light area indicates persistent carriers; the orange light area indicates intermittent carriers.

Overall, 65.5% (n = 57/87) of the participants carried *S*. *aureus* at least once during the six-month follow-up: 19.5% (n = 17) were persistent *S*. *aureus* carriers and 46.0% (n = 40) were intermittent carriers (Fig 2). There were 30 (34.5%) adults for whom *S*. *aureus* was never detected suggesting they were persistent non-carriers (S1 Fig).

Cefoxitin susceptibility test was performed for the 275 *S*. *aureus* isolates; all were susceptible displaying halos ranging from 23 to 38 mm. More than half (66.5%) of the *S*. *aureus* isolates displayed halos between ≥28 - ≤32 mm. No MRSA were detected.

## Discussion

We evaluated the prevalence of *S*. *aureus* and MRSA colonization among immunocompetent healthy Portuguese adults aged between 25–50 years old. We observed that c.a. two thirds (65.5%) of the individuals carried *S*. *aureus* at least once during the six months: 20% were persistent carriers and 46% were intermittent carriers. Our results, although not directly comparable, are in line with recent studies from other countries conducted among the general population. In a study conducted in Mexico, throat swabs were collected annually during six years from individuals aged 17–66 years old. The authors observed that 85.5% of the population carried *S*. *aureus* [24]. In a study conducted in Germany, nasal swabs were collected thrice in intervals of 6–8 months from individuals 7–97 years old. The proportion of *S*. *aureus* carriers was 40.9% [15, 25]. Collectively, these and other studies (for a detailed review see [26]) indicate that a significant proportion of the general population is regularly colonized with *S*. *aureus*.

Although we screened a substantial number of samples from three sites (526 samples for each), we did not find any MRSA carrier.

**Fig 3:**
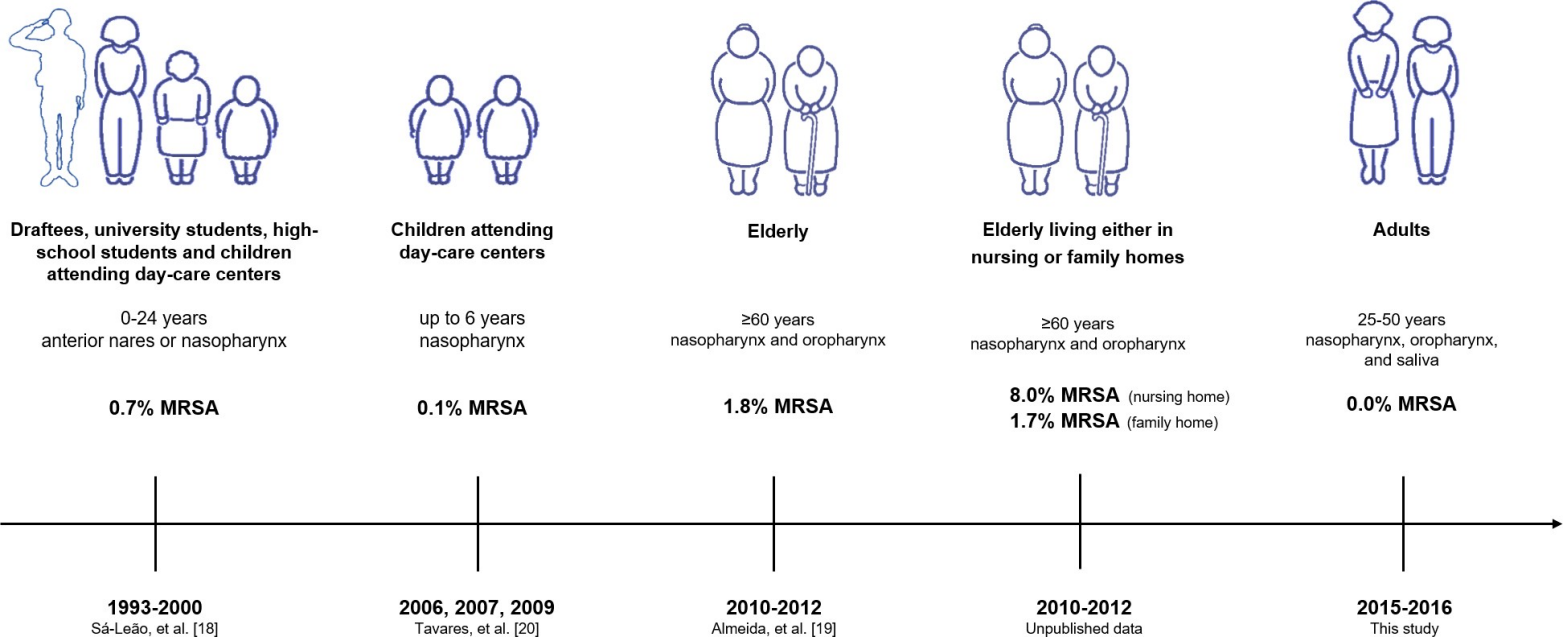
MRSA prevalence in the community in Portugal. Summary of studies performed among different populations of different age groups since 1993. For each study the age groups, sampling site and MRSA prevalence are indicated.

In Portugal, over the years, the study of MRSA in the community has spanned different groups of the population (summarized in Fig 3). In Portuguese children, previous findings reported very low rates of MRSA carriage, c.a. 0.2% [18, 20]. In the 1990’s a study of adolescents and young adults, namely, high-school students aged 13–16 years old, non-medical university students aged 21–24 years old, and draftees aged 17–22 years old, also reported very low MRSA colonization, <1% [18]. Two other studies, focusing on adults over 60 years of age, estimated MRSA carriage rates as <2% among individuals living in their family homes, and 5–8% among individuals living in nursing homes [19]. Of note, the few MRSA carriers identified in previous studies had, often, risk factors previously associated with MRSA carriage such as hospitalization in the months preceding the screening [18, 19]. Taken together, these results suggest that, although Portugal has a high prevalence of nosocomial MRSA, the prevalence of MRSA in the community is low among the healthy population without known risk factors. Although we have not investigated the reasons for this contrasting observations, they are in agreement with studies that suggest a fitness cost for HA-MRSA lineages [27, 28]. This cost seems to hamper dissemination of HA-MRSA in the absence of significant antibiotic pressure as was the case of the communities we studied which included mostly healthy individuals, not taking antibiotics, nor frequently exposed to health care institutions.

Our study has several limitations. First, we did not obtain samples from the anterior nares, which have been traditionally considered the preferential site of *S*. *aureus* colonization. However, we did obtain samples from different sites, in line with current recommendations to increase the detection of *S*. *aureus* and MRSA carriage [26, 29–32]. Our study supports this recommendation as the combined use of three sampling sites led to the detection of *S*. *aureus* in 34.4% of the sampling time-points while the use of a single sampling site would have detected *S*. *aureus* in a maximum of 20.5% of the samples (Fig 1). Secondly, we did not use an enrichment step, which some have found to increase detection of *S*. *aureus* and MRSA [12, 27]. However, this limitation was, in part, overcome by the collection of multiple samples from the same individual, which resulted in the detection of *S*. *aureus* in several individuals at several time-points, as described above and in line with findings from other studies. A third potential limitation of our study was the fact that typically a single colony with the characteristic properties of *S*. *aureus* was isolated and studied to evaluate whether the sample contained MRSA (evaluated through susceptibility to cefoxitin). In samples containing more than one strain of *S*. *aureus* this strategy would likely lead to the isolation of only the dominant strain. It is not impossible that MRSA present at a lower density might have been missed. Still, we consider this unlikely to have occurred with a frequency high enough to potentially change our main conclusions. Indeed, we have recently carried out a study among elderly adults where selective enrichment of samples followed by real-time PCR (qPCR) were used to increase the capacity to detect MRSA, and we found evidence that MRSA were rare and tended to be present as dominant population (unpublished data).

In conclusion, this study adds novel information about the MRSA scenario in the Portuguese community. Our results support that, in Portugal, MRSA does not seem to circulate among healthy adults without risk factors and therefore this age group does not constitute, at the current time, a reservoir of MRSA in the community.

## Supporting information

S1 Fig Representation of the 30 out of 87 participants that were never colonized with *S. aureus* during the six months of the study (non-carriers). Red circles represent negative samples; dotted circles represent expected samples (as per protocol) that were not obtained.
(TIF)
