# Supplementary figures and images for: Absence of methicillin-resistant Staphylococcus aureus colonization among immunocompetent healthy adults: Insights from a longitudinal study
Source: PLoS One. 2021 Jun 30;16(6):e0253739. doi: 10.1371/journal.pone.0253739 (PMC8244897; doi:10.1371/journal.pone.0253739)

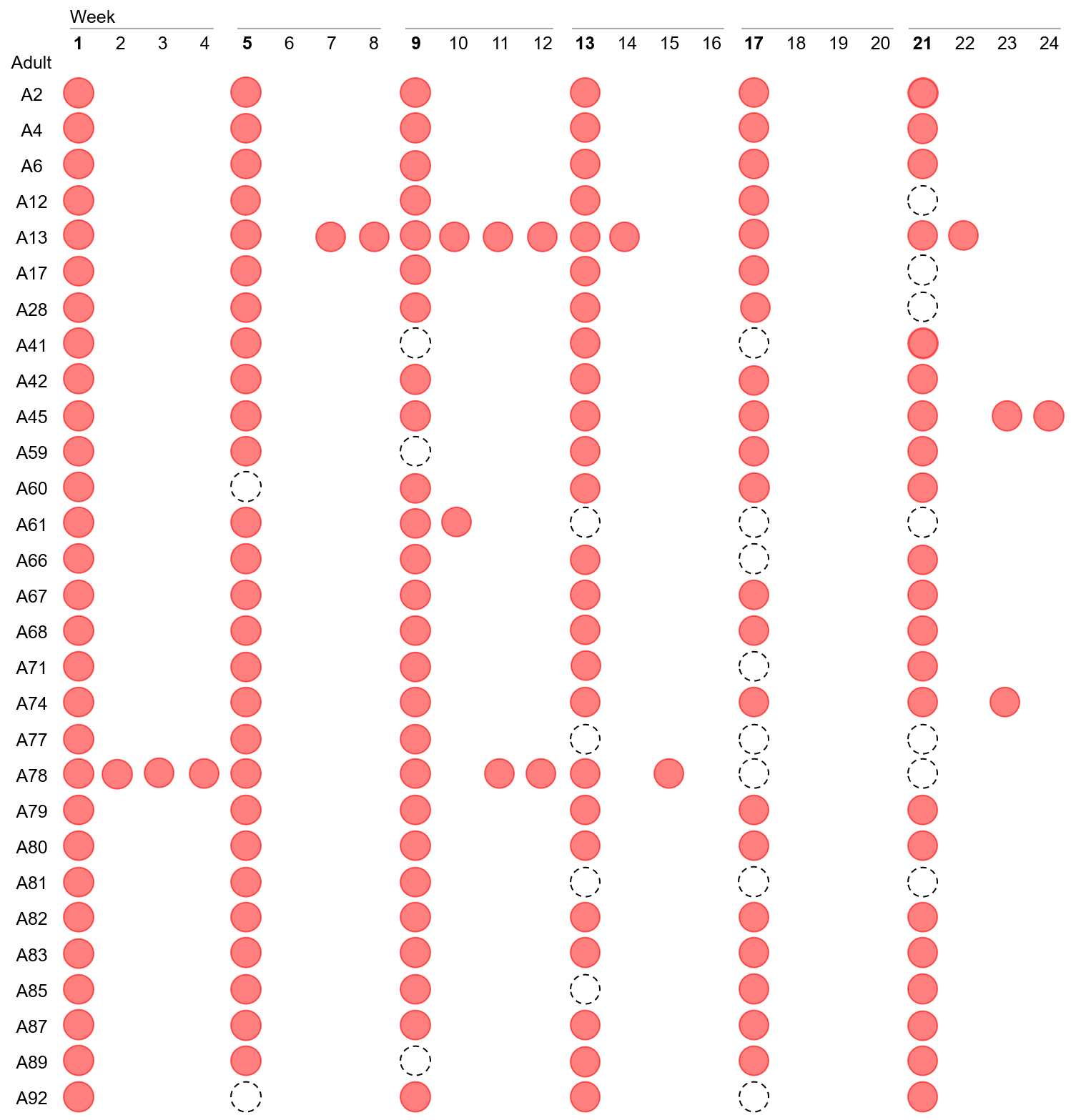

Supplement: S1 Fig — Red circles represent negative samples; dotted circles represent expected samples (as per protocol) that were not obtained. (TIF) [file pone.0253739.s001.tif]
